# Dietary carotenoid supplementation facilitates egg laying in a wild passerine

**DOI:** 10.1002/ece3.6250

**Published:** 2020-04-24

**Authors:** Jorge García‐Campa, Wendt Müller, Sonia González‐Braojos, Emilio García‐Juárez, Judith Morales

**Affiliations:** ^1^ Department of Evolutionary Ecology National Museum of Natural Sciences – Spanish National Research Council (CSIC) Madrid Spain; ^2^ Department of Biology Behavioural Ecology and Ecophysiology Group University of Antwerp Antwerp Belgium

**Keywords:** carotenoid allocation, egg production, maternal effects, self‐maintenance, trade‐off

## Abstract

During egg laying, females face a trade‐off between self‐maintenance and investment into current reproduction, since providing eggs with resources is energetically demanding, in particular if females lay one egg per day. However, the costs of egg laying not only relate to energetic requirements, but also depend on the availability of specific resources that are vital for egg production and embryonic development. One of these compounds are carotenoids, pigments with immuno‐stimulatory properties, which are crucial during embryonic development. In this study, we explore how carotenoid availability alleviates this trade‐off and facilitates egg laying in a small bird species, the blue tit (*Cyanistes caeruleus*). Blue tits have among the largest clutch size of all European passerines and they usually lay one egg per day, although laying interruptions are frequent. We performed a lutein supplementation experiment and measured potential consequences for egg laying capacity and egg quality. We found that lutein‐supplemented females had less laying interruptions and thus completed their clutch faster than control females. No effects of treatment were found on the onset of egg laying or clutch size. Experimentally enhanced carotenoid availability did not elevate yolk carotenoid levels or egg mass, but negatively affected eggshell thickness. Our results provide hence evidence on the limiting role of carotenoids during egg laying. However, the benefits of laying faster following lutein supplementation were counterbalanced by a lower accumulation of calcium in the eggshell. Thus, even though single components may constrain egg laying, it is the combined availability of a range of different resources which ultimately determines egg quality and thus embryonic development.

## Graphical abstract

This study provides the first evidence of limiting role of carotenoid during egg production. Experimentally enhanced carotenoid availability facilitated egg laying in blue tit females.

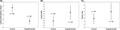

## 1 INTRODUCTION

Life‐history theory predicts that increased investment into current reproduction provides immediate fitness benefits via enhanced reproductive success, while it impinges at the same time on the amount of resources that can be maintained for self‐maintenance and thus for future reproduction (Stearns, 1992). In birds, females face this trade‐off between current and future reproduction among others when allocating resources to their eggs, as this increases offspring viability, but the costs of egg production compromise their rearing capacity and their prospects for future reproduction as well as survival (Monaghan, Nager, & Houston, 1998; Visser & Lessells, 2001). The high costs of egg production and the difficulties to maintain egg quality throughout laying are also reflected in the changes in egg composition along the egg sequence (Nager, Monaghan, & Houston, 2000; Williams & Miller, 2003).

Variation in egg composition along the laying sequence relates on the one hand to the energetic requirements for egg production that involve the acquisition of nutrients to be allocated to the eggs (Carey, 1996; Monaghan & Nager, 1997). Such energy or nutrient‐related effects on egg production have been studied in food supplementation experiments (Harrison et al., 2010; Ruffino, Salo, Koivisto, Banks, & Korpimaki, 2014). Indeed, providing females with more nutrients advanced the timing of reproduction (Vafidis et al., 2016) and had positive effects on clutch size (Korpimäki & Hakkarainen, 1991) or egg size (Ardia, Wasson, & Winkler, 2006). Laying capacity depends on the other hand on the availability of specific resources that are essential for embryonic development. One of these essential dietary micronutrients are carotenoids. These pigments are involved in a wide range of physiological processes, including the immune response (Pérez‐Rodriguez et al., 2008) and the transcription of antioxidant enzymes as well as metal‐binding proteins (Ben‐Dor et al., 2005; Cohen & McGraw, 2009). Carotenoids are crucial at early stages of development, since they reduce embryonic ROS damage (Surai & Speake, 1998) and enhance offspring immune system before (Surai, Speake, & Sparks, 2001) and after hatching (Haq, Bailey, & Chinnah, 1996; McWhinney, Bailey, & Panigrahy, 1989). Furthermore, they have a positive effect on offspring growth (Biard, Surai, & Møller, 2007) and influence the development of traits like plumage or beak coloration that play an important role in parent–offspring communication (Morales & Velando, 2013; Tschirren, Fitze, & Richner, 2005).

However, carotenoids cannot be endogenously synthetized by vertebrates and must be acquired from the diet (Olson & Owens, 1998). This implies that carotenoids could become limiting during highly demanding periods and that their use could be constrained by both their availability or by an individual's ability to find them. Thus, allocating carotenoids to eggs is expected to impose a cost for females (Karadas, Pappas, Surai, & Speake, 2005; McGraw, Adkins‐Regan, & Parker, 2005; Surai et al., 2001). Carotenoid demand for self‐maintenance processes is greater during the breeding season and, particularly, during egg laying, a period framed by a situation of high oxidative stress and immune‐depression (Hansell, 2005; Sheldon & Verhulst, 1996). This challenging period may—depending on clutch size—be extensive, since carotenoid acquisition and transfer to the eggs starts already days prior to egg laying (e.g., in birds, 5 days prior to laying; Surai et al., 2001). Egg quality is hence likely reduced under low carotenoid availability (Bortolotti, Negro, Surai, & Prieto, 2003). Indeed, previous studies have found that eggs laid by carotenoid supplemented females contained higher yolk carotenoid concentrations (e.g., Biard et al., 2007; Blount et al., 2002).

There is also some evidence that egg production per se could be limited by low carotenoid availability (Blount, Houston, & Møller, 2000; Blount, Houston, Surai, & Møller, 2004), potentially with negative effects on clutch size in conditions of low carotenoid availability (Eeva & Lehikoinen, 2010). This could be due to the fact that the allocation of carotenoids to eggs constrains self‐maintenance processes in the female, for example, their immune response (Blount, Metcalfe, Birkhead, & Surai, 2003; McGraw & Ardia, 2003). It is also possible that there is a minimum threshold of carotenoid availability during egg laying below which an egg cannot be laid. Furthermore, when carotenoids are limited, females may have to extend the laying period by lowering their egg laying rate or by interrupting egg laying, but this has as yet rarely been studied. In birds, females normally lay one egg per day until the clutch is completed, but laying interruptions of one or several days can occur (reviewed in Astheimer, 1985; see also Nilsson & Svensson, 1993). These interruptions have been reported to be more frequent under harsh conditions such as cold weather (Lessells, Dingemanse, & Both, 2002), high pollution (Eeva & Lehikoinen, 2010) and poor calcium availability (Bureš & Weidinger, 2003; Eeva & Lehikoinen, 2010; Graveland, 1996). Yet, such delays in the reproductive schedule may negatively affect fitness because they increase the time in which females and their clutches are vulnerable to predators (Milonoff, 1989), may decouple the timing for chick rearing with the peak of food availability (Durant et al., 2005), or may increase hatching asynchrony, if females start incubating before clutch completion (Magrath, 1990). Indeed, in our study population, females that perform laying interruptions have smaller chicks and lower fledging success (unpublished data; see also Stenning, 2018).

In this study, we explored whether lutein availability reduces the occurrence of egg laying interruptions in a small passerine, the blue tit (*Cyanistes caeruleus*). In this species, females make a substantial investment into their clutch, which can weigh up to 150% of their own body mass (Perrins & Birkhead, 1983; Stenning, 2018). As income breeders, blue tit females must acquire all the resources allocated to the clutch from their diet, for a period of up to 3 weeks. Maternal dietary carotenoids are likely of central importance as they are known to have significant implications for embryonic and posthatching development in blue tit (e.g., Biard et al., 2007; Surai & Speake, 1998; Valcu et al., 2019). Moreover, blue tit females supplemented with carotenoids at laying have been found to allocate more carotenoids to the egg yolk and to raise chicks with enhanced carotenoid‐based coloration (Biard, Surai, & Møller, 2005). We tested whether experimentally enhanced carotenoid availability prior and during laying affected the occurrence of laying interruptions, as well as clutch size and laying date, when controlling for environmental conditions. We predicted that carotenoid supplemented females would have less laying interruptions, may advance egg laying, and lay larger clutches. We also explored whether carotenoid supplementation influenced various aspects of egg quality like the amount of carotenoids in the yolk, as well as egg mass and shell thickness.

## 2 MATERIAL AND METHODS

### 2.1 General methods

The study was carried out in Miraflores de la Sierra, Community of Madrid, central Spain (40°48′N, 03°47′W) during the spring of 2017. We studied a nest‐box breeding blue tit population in a deciduous forest that is dominated by Pyrenean oak (*Quercus pyrenaica*). The blue tit is a territorial‐monogamous passerine that has one of the largest clutch sizes and the largest variation in clutch size among all European passerines (mean = 8.7; range from 3 to 22; Stenning, 2018). Blue tits in our population only raise one clutch per season. Eggs weigh on average 1.17 g (*n* = 1,001 eggs; Stenning, 2018), ranging from 0.97 to 1.41 g (Nilsson & Svensson, 1993). As many other passerines (Perrins, 1970), blue tit females lay one egg per day, but laying interruptions are frequent. In our study population, 33% of 64 un‐manipulated nests had laying interruptions; in a previous study (Stenning, 2018), 26% of 115 nests had laying interruptions. Laying interruptions are more abundant under environmental constraints and low food availability (Matthysen, Adriaensen, & Dhondt, 2011; Nilsson & Svensson, 1993; Stenning, 2018).

From the beginning of April onwards, nest boxes were visited every two days to determine the start of nest building. Blue tit nests are mainly made of moss, which is formed and lined with soft material such as hair and feathers. Nest building is mainly done by the female. As soon as the nest cup was defined (a hole in the moss not coated with soft material), which is typically 6.2 days prior to egg laying (range 1–14 days; unpublished data), we started lutein supplementation (see the following section). Lutein is a carotenoid pigment included in the group of xanthophylls. Lutein and zeaxanthin are the main carotenoids in bird plasma (>90%; McGraw, 2006) and in bird eggs (Surai et al., 2001), and are critical for offspring growth and feather color development (Morales & Velando, 2013; Surai et al., 2001; Tschirren et al., 2005).

Once egg laying started (first nest on 7th April and last nest on 27th April), nests were visited every second day and eggs were marked on the day they were found. Blue tit females lay one egg per day, so visiting nests every second day is sufficient to notice any laying interruptions. The fifth egg was collected immediately and substituted by a fake egg to prevent females from replacing it (collected between 11th April and 2nd June). We selected the fifth egg as this is likely most representative for the mean clutch level.

Lutein supplementation (see below) continued throughout egg laying and was stopped with the onset of incubation, defined as the first day that we noticed that the clutch was not cold. Both treatments were provided during the same range of time. For lutein‐supplemented females, first nest started on 1st April and last nest on 27th April. For control females, the first nest started also on 1st April and the last nest on 21st April. Treatment of the first female was randomly assigned, and thereafter, treatment was alternated. Lutein supplementation lasted on average 15.7 days prior to clutch completion (range: 9–23 days), as blue tit females tend to start incubating before the clutch is complete (Salvador, 2016; Stenning, 2018). A total of 92 nests were included in this experiment, and the average clutch size was 9.36 (*n* = 92, range 6–14). We supplemented control nests with bird fat‐containing nuts (*n* = 60) and lutein‐supplemented nests with the same amount of fat mixed with a lutein supplement (*n* = 32; see proportions and compounds in the “Manipulation of lutein availability” section).

We included the mean of minimum temperatures registered during egg laying for each nest, as ambient temperatures are known to affect egg laying (Lessells & Both, 2002; Matthysen et al., 2011). We obtained these data from a local weather station at the same altitude and nearby to the study area (Code = ESMAD2800000028792A—data available from ©Meteoclimatic.com). For analysis, we used the average of all minimum temperatures registered during the days that a female was laying.

In this study, we did not include hatching date effects due to a second experimental design in which we cross‐fostered clutches two days before the expected hatching date.

### 2.2 Manipulation of lutein availability

Once the nest cup was defined but not lined, we put a transparent plastic feeder into the nest box (2.5 × 4.5 × 4.5 cm), pinned to the inner back nest‐box wall. We chose this stage of nest building to ensure that the nest owners would continue breeding, since most nest usurpations occur at earlier stages. Nests were sequentially assigned to either a control group or to a lutein‐supplemented group. Initially, we created 60 control nests and 32 lutein‐supplemented nests. This unbalance between treatments was created in the context of a second experiment mentioned above, in which we needed twice the number of control nests than lutein‐supplemented nests. In the present study, we nevertheless used all data available. One nest was deserted during food supplementation as result of a nest usurpation by another blue tit pair.

Lutein was provided every second day, and each dosage consisted of 50 mg of Versele Laga Yel‐lux Oropharma (lutein 8,000 mg/kg), which corresponds to 0.4 mg of lutein. According to Partali, Liaaen‐Jensen, Slagsvold, and Lifjeld (1987), one lepidopteran larvae in the natural diet of blue tits contains on average 5.3 µg of lutein, and thus, the dosage used would correspond, approximately, to 75 prey items. To our knowledge, no studies on free‐living blue tits have focused on the amount of lepidopteran larvae that laying females eat on average. However, blue tits chicks consume on average 100 lepidopteran larvae per day during development (Gibb & Betts, 1963). Thus, we used the natural amount of food consumed by nestlings as proxy of the daily amount consumed by laying females and our experimental manipulation every two days lies within the natural range of the species—at least for young. Each lutein dose was mixed with 5 g of commercial fat with nuts (GRANA Oryx). Control nests received the same amount of fat but without lutein. Each lutein dose was weighted in advance with a digital analytical balance (accuracy 0.001 mg) and stored in Eppendorf tubes at 4°C in the dark to prevent oxidation. Lutein doses were mixed with the corresponding amount of fat just before supplementation at the nest.

We supplemented nests until the female started incubation. At each visit, we weighed the amount of food that remained at the feeder using a Pesola spring balance (to the nearest 0.01 g), cleaned the feeder and refilled it with 5 g with the corresponding treatment. We then calculated the total amount of food consumed over the experiment, which we used in the statistical analyses. We also noted the first day when a minimum of 0.5 g of food was consumed, in order to estimate the number of days it took a given female to start consuming food (hereafter also termed “food neophobia”). We established this threshold based on a pilot study, where we observed that intact food may weigh between 0.1 and 0.5 g less than the day before, probably because it loses water. In a subsample of nests (*n* = 15), we confirmed by means of video recordings that males rarely visited the nest box, and thus, we assume that any food that disappeared was consumed by the female.

In this experiment, we have used Yel‐Lux as lutein supplement. This product has been used in previous lutein supplementation studies in order to increase carotenoid availability (e.g., Hargitai, Boross, Nyiri, & Eke, 2016) and has been traditionally used by bird breeders (Langner & Bulk, 2016). Yel‐Lux is a flower extract from African marigold (*Tagetes erecta L.*) and also contains calcium lactate, ethoxyquin, and dextrose in unknown amounts as preservatives of lutein. The composition is very similar to other previously used dietary carotenoid supplements like ORO GLO (e.g., Casagrande, Pinxten, Zaid, & Eens, 2014; van Hout, Eens, & Pinxten, 2011). Calcium lactate, also known as E327, is used as preservative in the alimentary and medical industry to stabilize the structure of products during processing. We consider it unlikely that it affected the outcome of our results, given that yel‐lux is not used as a calcium supplement but as a lutein supplement. This is supported by the result that lutein‐supplemented females did not allocate more calcium to the eggs, rather, they decreased it (see Results section). Ethoxyquin is supposed to decrease circulating cholesterol and to produce low concentrations of proteins in blood (proteinuria; Hill, 1966). However, previous studies using lutein supplementation in birds did not find effects on circulating cholesterol or an increase of albumin levels in blood (Casagrande et al., 2014). Finally, because the control treatment consisted on bird fat with seeds, which are rich in simple sugars, we can rule out a strong effect of dextrose.

### 2.3 Egg measurements

We collected the 5th egg on the day of laying (*n* = 80 clutches; 51 from control nests and 29 from lutein‐supplemented nests) and weighed it in the field to the nearest 0.01 g. We registered egg mass in 79 eggs because one egg of the lutein‐supplemented group broke during the egg collection. Eggs were kept cool and within the same day they were stored at −80°C until the analyses. For the analysis, we defrosted all eggs on the same day and separated the yolk, the albumen, and the eggshell, and weighed the yolk using a digital analytical balance (accuracy 0.001 g). We added to the yolk twice the volume of water and vortexed this mixture at the highest speed for 1 min. Yolks were again stored at −80°C until the following day, when they were defrosted again for carotenoid analyses. We could use 76 samples for carotenoid analysis (48 from control nests and 28 from lutein‐supplemented nests) because yolk and albumen were mixed in a few samples after defrosting.

From that yolk–water mixture, 100 μl was transferred to a new Eppendorf and 400 μl of pure ethanol was added. The mixture was then centrifuged at 1,500 G during 5 min at room temperature, and the aqueous phase was transferred to an Eppendorf tube. Optical density was obtained at 450 nm using a Synergy™ HT Multi‐model Microplate Reader (BioTek^®^ Instruments, Inc.). Carotenoid concentrations were obtained from a lutein analytical standard (Sigma‐Aldrich^®^). Plate number was registered for each sample and controlled for in statistical analyses. The analysis was repeated in duplicate in all cases (coefficient of variation = 2.95) In a subsample of eggs (*n* = 30), we also analyzed lutein concentration by high‐performance liquid chromatography (HPLC) with ethanol extraction following Alonso‐Álvarez et al. (2004). Both measurements were positively correlated (*r*_30_ = 0.41, *p* = .02). We calculated yolk carotenoid content by multiplying carotenoid concentration and yolk mass.

Eggshell thickness of all the 80 collected eggs was measured using a digital tube micrometer (Mitutoyo Ip‐65) with ballpoint ends and precision of 0.001 mm, following Morales et al. (2013). We took nine measures per egg (if possible, 3 in each of the following eggshell locations: blunt end, sharp end, and equator). When it was not possible to identify the specific location of the eggshell, it was categorized as “indeterminate.” Measures at the different locations showed high repeatability; thus, measures were highly consistent (blunt end: *r* = .8, *F* = 10.7, *p* < .001; sharp end: *r* = .8, *F* = 12.7, *p* < .001; equator: *r* = .8, *F* = 17.3, *p* < .001; indeterminate: *r* = .8, *F* = 4.4, *p* < .001; all measures pooled: *r* = .7, *F*_1,79_ = 21.4, *p* < .001). Thus, we calculated the mean of each eggshell location. We clearly identified equator location for 48 shells, 33 shells for sharp end location, and 24 for shells from blunt end location.

### 2.4 Statistical analyses

First, we tested whether the total amount of food consumed and food neophobia (i.e., the number of days it took a given female to start consuming food) differed between the control and the lutein‐supplemented group. Food neophobia was analyzed using a generalized linear model (GLZ) with Poisson distribution and a log link function, including treatment as a predictor variable. The total amount of food consumed was analyzed using a general linear model (GLM), including treatment as predictor variable.

Second, we tested the effect of treatment on laying capacity variables (laying date, number of laying interruptions, and clutch size) and egg quality variables (egg mass, yolk carotenoid content, and eggshell thickness). Laying date and egg mass were analyzed using GLMs. The number of laying interruptions and clutch size were analyzed using a GLZ with Poisson distribution. In these models, we included treatment, the total amount of food consumed, and the interaction between both as predictor variables. We also included clutch size, the minimum temperature, and their interactions with treatment as predictor variables in the analysis of laying interruptions.

Finally, yolk carotenoid content and shell thickness were analyzed using mixed models. For the yolk carotenoid content, we included lab plate (three categories) as a random factor. For eggshell thickness, we included nest id as random factor in order to account for repeated measures at different shell locations. For both models, we included the following fixed predictor variables: treatment, the total amount of food consumed, eggshell location in the case of eggshell thickness (blunt end, sharp end, or equator) and all double interactions with treatment.

We used SAS 9.4 (SAS Inst.) for all statistical analyses. Backward elimination of nonsignificant interactions (*α* = 0.05) was used to acquire minimal models. The models were checked for residual normality with a Shapiro normality test. In the text, we report minimal models after backward elimination (Tables 1 and 2), while full initial models are shown in Tables S1 and S2.

**TABLE 1:** Final minimal models after backward elimination of nonsignificant interactions showing the effect of carotenoid supplementation on laying capacity and food consumption

	Laying interruptions (days)	Laying date	Clutch size	Total amount of food consumed (g)	Food neophobia (days)
Intercept	coef = 1.58 ± 1.24	coef = 14.61 ± 0.79	coef = 2.22 ± 0.07	coef = 10.16 ± 1.39	coef = 0.89 ± 0.18
Treatment (control)	coef = 1.08 ± 0.50 $\chi_{1}^{2}$ = 5.77 ***p* = .016**	coef = −1.04 ± 0.78 *F*_1,89_ = 1.76 *p* = .19	coef = −0.01 ± 0.07 $\chi_{1}^{2}$ = 0.04 *p* = .85	coef = −2.94 ± 1.72 *F*_1,90_ = 2.94 *p* = .090	coef* = *−0.02 ± 0.22 $\chi_{1}^{2}$ = 0.01 *p = *.92
Total amount of food consumed (g)	coef = −0.04 ± 0.03 $\chi_{1}^{2}$ = 1.73 *p* = .19	coef = −0.11 ± 0.05 *F*_1,89_ = 5.35 ***p* = .023**	coef = 0.003 ± 0.004 $\chi_{1}^{2}$ = 0.50 *p* = .48		
Minimum Temperature (°C)	coef = −0.18 ± 0.10 $\chi_{1}^{2}$ = 3.52 *p* = .061				
Clutch Size	coef = −0.14 ± 0.12 $\chi_{1}^{2}$ = 1.36 *p* = .24				

Note

General lineal models were performed for laying date and total food consumed. Laying interruptions, food neophobia, and clutch size models were performed using generalized lineal models. Coefficients are shown for control nests. Significant differences are marked in bold.

**TABLE 2:** Final minimal models after backward elimination of nonsignificant interactions showing the effects of treatment on egg quality

	Egg mass (g)	Yolk carotenoid content (µg)	Eggshell thickness (mm)
Intercept	coef = 1.10 ± 0.02	coef = 3.44 ± 0.38	coef = 0.06 ± 0.001
Treatment (control)	coef = 0.01 ± 0.02 *F*_1,77_ = 0.77 *p* = .38	coef = −0.06 ± 0.25 *F*_1,71_ = 0.05 *p* = .82	coef = 0.002 ± 0.001 *F*_1,153_ = 7.55 ***p* = .0067**
Total amount of food consumed (g)	coef = 0.0001 ± 0.001 *F*_1,77_ = 0.02 *p* = .90	coef = 0.02 ± 0.02 *F*_1,71_ = 1.43 *p* = .24	coef = −0.00002 ± 0.0001 *F*_1,153_ = 0.16 *p* = .69
Eggshell location (sharp end)			coef = 0.001 ± 0.001 *F*_5,153_ = 2.77 ***p* = .020**

Note

A general lineal model was performed for egg mass. Mixed models were performed for eggshell thickness and yolk carotenoid content. Coefficients are shown for control nests and sharp end locations. Significant differences are highlighted in bold.

All tests were conducted by using Type III sums of squares. This adjustment is based on unweighted marginal means in order to accommodate analyses with unequal sample sizes. This approach avoids to estimate group means with different levels of precision and is much more robust to violations of, for example, homogeneity of variances (Quinn & Keough, 2002).

## 3 RESULTS

### 3.1 Total amount of food and food neophobia

Treatment did not significantly affect the total amount of food consumed (*p* = .090; Table 1). There was no effect of treatment on food neophobia either (*p* = .92; Table 1). On average, females started to eat 2.4 days (2.43 days for lutein and 2.38 days for control females) after the feeder was placed into nest box (range 1–13 days). None of the interactions with treatment was significant (Table S1).

### 3.2 Egg laying capacity

**FIGURE 1:**
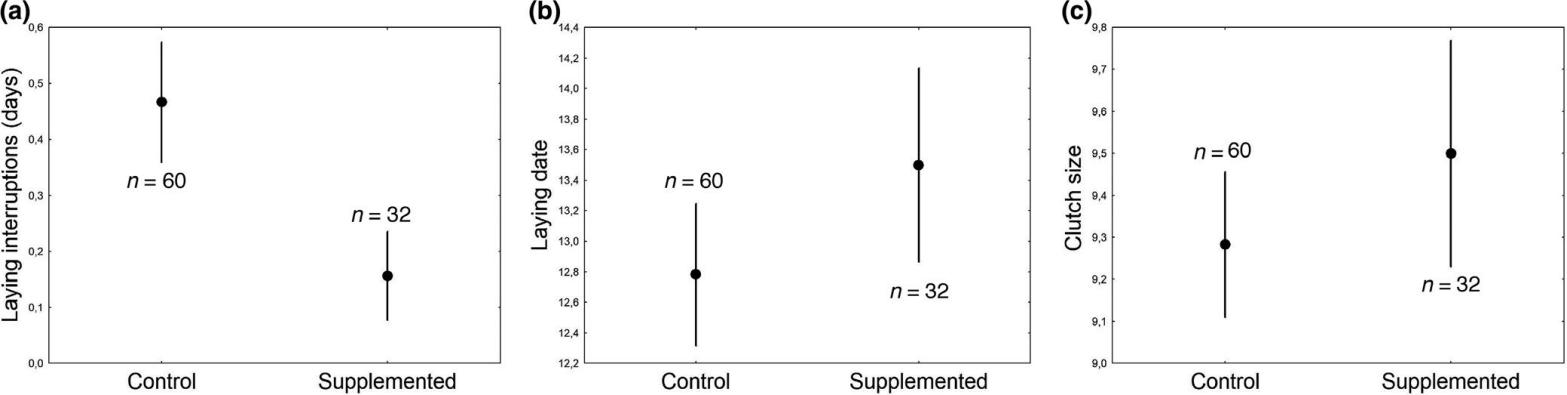
Laying capacity of control and lutein‐supplemented blue tit females (*Cyanistes caeruleus*): (a) Number of laying interruptions during egg laying that females had on average (days); (b) laying date according to the Julian calendar (1 = 1st April); (c) clutch size. Error bars denote standard errors (mean ± *SE*). Sample sizes for each treatment are shown

There was no treatment effect on the onset of egg laying (*p* = .19; Table 1; Figure 1b) or on clutch size (*p* = .85; Table 1; Figure 1c). Females which consumed more food during treatment started laying eggs before females that consumed less food (*p* = .023; Table 1). Lutein‐supplemented females had less laying interruptions than control females *p* = .016; Table 1; Figure 1a). On average, 30% of control females had laying interruptions (*n* = 18 of 60) and 12.5% of lutein‐supplemented females (*n* = 4 of 32). Lower minimum temperatures during egg laying tended to be associated with more laying interruptions (*p* = .061; Table 1). All interactions with treatment were not significant (see Table S1).

### 3.3 Egg quality

**FIGURE 2:**
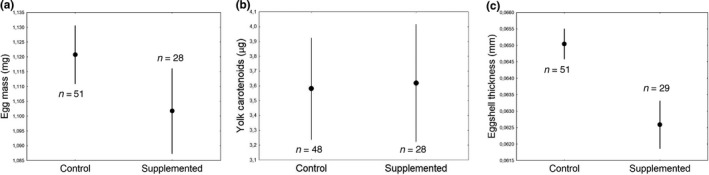
Egg quality measures (mean ± *SE*) of control and lutein‐supplemented blue tit females (*Cyanistes caeruleus*): (a) Egg mass of the 5th collected egg (g); (b) yolk carotenoid content of the 5th collected (µg); (c) eggshell thickness of the 5th collected (mm); values of shell thickness are the mean measured in all locations pooled (blunt end, sharp end, and equator). Sample sizes for each treatment are shown

No effects of treatment (*p* = .38; Figure 2a) or of the total amount of food consumed (*p* = .90) were found on egg mass (Table 2). The interactions with treatment were not significant (Table S2).

The yolk carotenoid content of the fifth egg was not affected by treatment *p* = .82; Table 2; Figure 2b) or by the total amount of food consumed (*p* = .24; Table 2). The interaction between total amount of food consumed and treatment had no significant effect (Table S2).

Lutein‐supplemented females laid eggs with thinner eggshells than control females (*p* = .0067; Figure 2c). On average, lutein‐supplemented females laid eggs 4.5% thinner than control females. Eggshell thickness did not vary with the total amount of food consumed (Table 2). Eggshell thickness depended on the eggshell location (*p* = .020; see also Table 2), sharp end locations being thicker than blunt end locations (least square difference: *p* = .030), but not to equator locations (*p* = .12). None of the interactions with treatment was significant (Table S2).

## 4 DISCUSSION

We hypothesized that dietary carotenoid availability during egg laying is likely of central importance for female blue tits. Indeed, experimentally enhanced carotenoid availability allowed lutein‐supplemented females to have less laying interruptions. However, this came at a potential cost as lutein‐supplemented females laid eggs with thinner eggshells than control females. Intriguingly, other aspects of a female's laying capacity or the allocation of carotenoids to the yolk were not affected. The potential causes of these findings are discussed below.

### 4.1 Egg laying capacity

As expected, we did find an effect of treatment on the number of laying interruptions. Lutein‐supplemented females completed their clutch faster than control females (70% of control females laid one egg per day, against 87.5% of lutein‐supplemented females), while clutch size was similar in both treatments. This is the first evidence for effects of lutein availability on laying interruptions. We hypothesized that lutein is limited under natural conditions and is required by females for both egg laying and self‐maintenance processes. Thus, the lutein supplement might have allowed experimental females to finish their clutch faster than control females. Nonetheless, we did not find differences on yolk carotenoid content between treatments, which suggests that carotenoids were allocated to the females’ self‐maintenance and that costs in terms of resource limitation are mostly paid by the offspring. Previous studies in blue tits showed that laying interruptions occur frequently, probably as a consequence of environmental constraints (Janssens, Dauwe, Pinxten, & Eens, 2003). In our study, the effect of lutein remained even after controlling for the amount of food consumed and for the effects of low minimum temperatures during egg laying. However, we did not control for female body mass during egg laying (not measured at this stage due to the risk of nest abandonment), which could have been affected for food supplementation. Thus, we cannot discard an effect of female body mass on egg laying interruptions. Finally, although nonsignificant, the negative trend that we found regarding temperature supports previous studies in other blue tit populations (Yom‐Tov & Wright, 1993).

Longer laying times can have serious consequences for parental rearing capacity (Perrins, 1970), egg viability (Milonoff, 1989), and nestling survival (Hochachka, 1990; Martin & Hannon, 1987; Nilsson, 1990). The latter may arise via increased hatching asynchrony, if incubation starts before the clutch is completed, which disadvantages later hatching chicks (Magrath, 1990). However, the occurrence of laying interruptions has also been proposed as a mechanism to delay the breeding schedule in order to better match the peak of caterpillar availability rather than representing a physiological constraint (Cresswell & McCleery, 2003; Tomás, 2015). Our results are in line with this idea if increased availability of carotenoids serves as an indicator of the proximity of the caterpillar peak. However, even though laying interruptions in our study prolonged laying by 1.5 days (range 1–3) on average, it seems unlikely that this delay would provide a marked difference in the (mis)match with the caterpillar peak. Thus, the possibility of a physiological constraint based on carotenoid limitation at laying is a more likely and mutually nonexclusive explanation for our findings. Lutein treatment had no effects on laying date, while several studies have shown a positive effect of food supplementation on the timing of reproduction (Martin & Hannon, 1987; Meijer & Drent, 2008; Robb, McDonald, Chamberlain, & Bearhop, 2008). Yet, these effects may relate to caloric restrictions and not to the availability of specific nutrients. Due to our experimental design, we did not expect large effects on laying date. In the current study, feeding both control and lutein‐supplemented females with bird fat allowed us to avoid metabolic or energetic effects not related to female carotenoid requirements and to focus on the role of carotenoids.

There was no effect on clutch size (see also Harrison et al., 2010), despite the fact that previous studies showed that carotenoids may be limiting for egg production (see Biard et al., 2005, using a much higher lutein dose). In the study year, the average clutch size in control and experimental females was 9.36 ± 0.15 (range: 6–14, *n* = 92), which does not differ from the following two years in which females were not supplemented (9.56 ± 0.14, range 4–15, *n* = 206). Thus, it seems that the plasticity in clutch size is limited at least in the study population and does not depend greatly in food availability or in other specific substances (see also Moreno & Carlson, 1989 in the pied flycatcher *Ficedula hypoleuca*).

### 4.2 Egg quality

Unexpectedly, we did not find differences in yolk carotenoid content between our treatments. This is in contrast to previous studies showing effects of carotenoid supplementation on yolk carotenoid content in wild blue tits (Biard et al., 2005; Blount et al., 2002) and in other captive bird species (Bortolotti et al., 2003; Surai & Sparks, 2001; Surai & Speake, 1998). However, these studies substantially manipulated carotenoids potentially various magnitude orders above the natural range (more than 100 times the daily consumption; daily amount of lutein supplemented: 500 mg in Biard et al., 2005; 1.75 in Remeš, Krist, Bertacche, & Stradi, 2007). Here, we supplied females with carotenoids within the biological range (0.4 mg in this study; see Partali et al., 1987). Our results suggest that females used the extra carotenoids for other physiological functions related to self‐maintenance and to enhance the laying capacity (Hargitai et al., 2006; Isaksson, Johansson, & Andersson, 2008; Navara, Badyaev, Mendonça, & Hill, 2006). Thus, in the context of a trade‐off between allocation to eggs and to self‐maintenance (Giordano, Groothuis, & Tschirren, 2014; Morales, Velando, & Moreno, 2008), self‐maintenance is prioritized until carotenoid supplementation goes beyond the levels required by the female, when it may indeed be reflected in higher yolk carotenoid contents. It has to be considered that carotenoid transfer to the follicle occurs each 24 hr (Salvante & Williams, 2002), and, thus, if females had allocated more carotenoids to the yolk, we should have detected differences between treatments in the fifth collected egg. Thus, the lack of an effect on yolk carotenoid content in our study is more likely explained by females using carotenoids for self‐maintenance functions than being an artifact of our methodology.

We did not find effects of treatment on egg mass, but both control and lutein‐supplemented females received additional resources that could have been used for egg formation.

We found a treatment effect on eggshell thickness, with lutein‐supplemented females laying thinner eggs than control females. One explanation is that laying interruptions allow females to accumulate calcium resources, which is reflected in increased shell thickness. Thus, carotenoid supplementation facilitated egg laying, but fewer laying interruptions during laying prevented the deposition of calcium in the eggshell, either because lutein‐supplemented females had less time for foraging on calcium‐rich resources between eggs (note that short foraging bouts result in increased quantities of calcium; Flint, Fowler, Bottitta, & Schamber, 1998), and that the lack of calcium makes laying interruptions more frequent in other species (e.g., pied flycarcher, *Ficedula hypoleuca*; Bureš & Weidinger, 2003) or because their eggs stayed—on average—less time in the oviduct compared to control eggs. Eggshell is the physical barrier between the embryo and the environment, and its thickness has profound consequences on incubation efficiency by heat transference (Soliman, Rizk, & Brake, 1994), microbial infection (D'Alba, Jones, Badawy, Eliason, & Shawkey, 2014), water loss (Drent & Woldendorp, 1989), and egg viability (Mellanby, 1992). Therefore, our results suggest that an effect of laying faster could be a lower accumulation of calcium in the eggshell, which indicates that the combined availability of different resources determines egg quality.

Several studies have criticized the antioxidant capacity of carotenoids, especially for xanthophylls as lutein (reviewed in Costantini & Møller, 2008), while carotenoids nevertheless signal the oxidative status of individuals—because their structure and function are extremely sensible to ROS damage (Bertrand et al., 2006; Pérez‐Rodriguez et al., 2008). However, carotenoids also have immuno‐stimulatory properties, that is, regulating thymocyte activation (Garbe, Buck, & Hämmerling, 1992), the expression of immune‐related genes (Geissmann et al., 2003), and proteins involved in cell‐to‐cell communication (Basu, Vecchio, Flider, & Orthoefer, 2001). In addition, carotenoids play a role in promoting the transcription of antioxidant enzymes and metal‐binding proteins (reviewed in Pérez‐Rodríguez, 2009). All of these properties are likely relevant for the developing embryo and may also be a reason as to why egg yolk contains such high carotenoid concentration. As a final remark, in this experiment we use Yel‐Lux as a lutein supplement, which contains a number of additional compounds, which should be taken into account for the interpretation, even though is unlikely that it affected our results (see Material and Methods).

To conclude, this study provides the first evidence that experimentally enhanced carotenoid availability allowed blue tit females to complete their clutch faster. This suggests that carotenoids are a limiting resource in the blue tit, a species that lays large clutches in a very short time interval. Yet, lutein supplementation did not lead to higher yolk carotenoid content, which suggests that females used the extra carotenoids for self‐maintenance or to enhance their laying capacities. The supplementation of a single compound, here lutein, also revealed a trade‐off between laying in short sequence and calcium deposition in the eggshell, since lutein‐supplemented females laid eggs with thinner shells. To summarize, our results emphasize the limiting role that carotenoids play for blue tit females during the egg production.

## CONFLICT OF INTEREST

Authors declare that they have no conflict of interest.

## AUTHOR CONTRIBUTION

**Jorge García‐Campa:** Data curation (equal); Formal analysis (equal); Investigation (equal); Methodology (equal); Writing‐original draft (equal); Writing‐review & editing (equal). **Wendt Müller:** Conceptualization (equal); Formal analysis (equal); Investigation (equal); Methodology (equal); Supervision (equal); Writing‐review & editing (equal). **Sonia González‐Braojos:** Investigation (equal); Methodology (equal); Visualization (equal). **Emilio García‐Juárez:** Methodology (equal); Visualization (equal). **Judith Morales:** Conceptualization (equal); Data curation (equal); Formal analysis (equal); Funding acquisition (equal); Investigation (equal); Methodology (equal); Project administration (equal); Resources (equal); Supervision (equal); Visualization (equal); Writing‐review & editing (equal). 

### Open Research Badges

This article has been awarded <Open Materials, Open Data, Preregistered Research Designs> Badges. All materials and data are publicly accessible via the Open Science Framework at https://doi.org/10.5061/dryad.p5hqbzkm8; https://doi.org/10.1101/792234; https://doi.org/10.1101/792234.

## Supporting information

Tables S1‐S2

## Data Availability

Data available from Dryad Digital Repository (García‐Campa, Müller, González‐Braojos, García‐Juárez, & Morales, 2021: https://doi.org/10.5061/dryad.p5hqbzkm8).
