# Supplementary material for: Dietary carotenoid supplementation facilitates egg laying in a wild passerine
Source: Ecol Evol. 2020 Apr 24;10(11):4968–78. doi: 10.1002/ece3.6250 (PMC7297774; doi:10.1002/ece3.6250)
Supplement: Supplementary file 1 — Tables S1‐S2 [file ECE3-10-4968-s001.docx]

**Dietary carotenoid supplementation facilitates egg laying in a wild passerine**

Jorge García-Campa^a*^, Wendt Müller^b^, Sonia González-Braojos^a^, Emilio García-Juárez^a^ & Judith Morales^a^

^a^ Department of Evolutionary Ecology, National Museum of Natural Sciences – Spanish National Research Council (CSIC). c/ José Gutiérrez Abascal 2, 28006 Madrid, Spain. ^b^ Department of Biology, Behavioural Ecology and Ecophysiology Researchgroup, University of Antwerp, Universiteitsplein 1, 2610 Antwerp, Wilrijk, Belgium *Correspondence: [jgarciacampa@gmail.com](mailto:jgarciacampa@gmail.com)

Table 1. Full models before backward deletion of non-significant interactions showing the effect of carotenoid supplementation on laying capacity and food consumption. General lineal models were performed for laying date and total food consumed. Laying interruptions, clutch size and food neophobia models were performed using Generalized lineal models. Coefficients are shown for control nests. Significant differences are marked in bold.

|  | *Laying interruptions (days)* | *Laying date* | *Clutch Size* | *Total amount of food consumed (g)* | *Food neophobia*  *(days)* |
| --- | --- | --- | --- | --- | --- |
| *Intercept* | *coef* = -0.36 ± 3.41 | *coef* = 15.21 ± 1.05 | *coef* = 2.18 ± 0.10 | *coef* = -10.16 ± 1.39 | *coef* = 0.89 ± 0.18 |
| *Treatment*  *(control)* | *coef* = 3.05 ± 3.64  *χ*²_1_ = 0.73  *P* = 0.39 | *coef* = -1.84 ± 1.22  *F*_1,88_ = 2.28  *P* = 0.14 | *coef* = 0.05 ± 0.11  *χ*²_1_ = 0.16  *P* = 0.69 | *coef* = -2.94 ± 1.72  *F*_1,90_ = 2.94  *P* = 0.090 | *coef =* -0.02 ± 0.22  *χ²*_1_ *=* 0.01  *P =* 0.92 |
| *Total amount of food consumed (g)* | *coef* = -0.26 ± 0.16  *χ²*_1_ = 5.19  ***P* = 0.023** | *coef* = -0.17 ± 0.08  *F*_1,88_ = 6.07  ***P* = 0.016** | *coef* = 0.007 ± 0.008  *χ²*_1_ = 0.81  *P* = 0.37 |  |  |
| *Clutch Size* | *coef* = -0.03 ± 0.33  *χ²*_1_ = 0.19  *P* = 0.67 |  |  |  |  |
| *Minimum Temperature*  *(⁰C)* | *coef* = 0.03 ± 0.28  *χ²*_1_ = 0.40  *P* = 0.53 |  |  |  |  |
| *Total food consumed**  *Treatment* | *coef* = 0.24 ± 0.16  *χ²*_1_ = 3.57  *P* = 0.059 | *coef* = 0.09 ± 0.10  *F*_1,88_ = 0.74  ***P* = 0.39** | *coef* = -0.006 ± 0.009  *χ²*_1_ = 0.46  *P* = 0.50 |  |  |
| *Clutch Size**  *Treatment* | *coef* = -0.10 ± 0.36  *χ²*_1_ = 0.07  *P* = 0.79 |  |  |  |  |
| *Minimum Temperature *Treatment* | *coef* = -0.25 ± 0.30  *χ²*_1_ = 0.66  *P* = 0.42 |  |  |  |  |

Table 2. Full models before backward deletion of non-significant interactions showing the effects of treatment on egg quality. General lineal model was performed for egg mass. Mixed model were performed for yolk carotenoid content and eggshell thickness. Coefficients are shown for control nests and sharp end location. Significant differences are marked in bold.

|  | *Egg mass*  *(g)* | *Yolk carotenoids*  *(µg)* | *Eggshell thickness*  *(mm)* |
| --- | --- | --- | --- |
| *Intercept* | *coef* = 1.12 ± 0.03 | *coef* = 3.65 ± 0.46 | *coef* = 0.06 ± 0.001 |
| *Treatment*  *(control)* | *coef* = -0.007 ± 0.03  *F*_1,76_ = 0.06  *P* = 0.80 | *coef* = -0.33 ± 0.42  *F*_1,70_ = 0.62  *P* = 0.43 | *coef* = 0.002 ± 0.002  *F*_1,147_ = 0.93  *P* = 0.34 |
| *Total amount of food consumed (g)* | *coef* = -0.001 ± 0.002  *F*_1,76_ = 0.08  *P* = 0.77 | *coef* = -0.001 ± 0.03  *F*_1,70_ = 0.55  *P* = 0.46 | *coef* = -0.0001 ± 0.0001  *F*_1,147_ = 0.48  *P* = 0.49 |
| *Eggshell location*  *(sharp end)* |  |  | *coef* = 0.003 ± 0.001  *F*_1,147_ = 3.49  ***P* = 0.0052** |
| *Total food consumed**  *Treatment* | *coef* = 0.002 ± 0.002  *F*_1,76_ = 0.98  *P* = 0.33 |  | *coef* = 0.0001 ± 0.0001  *F*_5,147_ = 0.57  *P* = 0.45 |
| *Eggshell location*  ** Treatment* |  | *coef* = 0.03 ± 0.03  *F*_1,70_ = 0.66  *P* = 0.42 | *coef* = -0.002 ± 0.002  *F*_5,147_ = 0.95  *P* = 0.45 |
|  |  |  |  |
